# 2895. Expanding PrEP by Embedding Navigators in High STI Testing Clinical Sites

**DOI:** 10.1093/ofid/ofad500.166

**Published:** 2023-11-27

**Authors:** R Scott Braithwaite, Robert Pitts, Farzana Kapadia, Kaoon Ban

**Affiliations:** NYU Langone Health, New York City, New York; NYC H+H/Bellevue, New York City, NY; NYU School of Global Public Health, New York, New York; NYU Langone School of Medicine, New York, New York

## Abstract

**Background:**

Opportunities for PrEP continue to be missed in key populations. We established a PrEP navigation program (“SNAPS”) with the goals to (1) increase PrEP uptake among groups disproportionately impacted by HIV and (2) preserve and improve PrEP adherence in an NYC safety-net hospital setting.

**Table 1:**
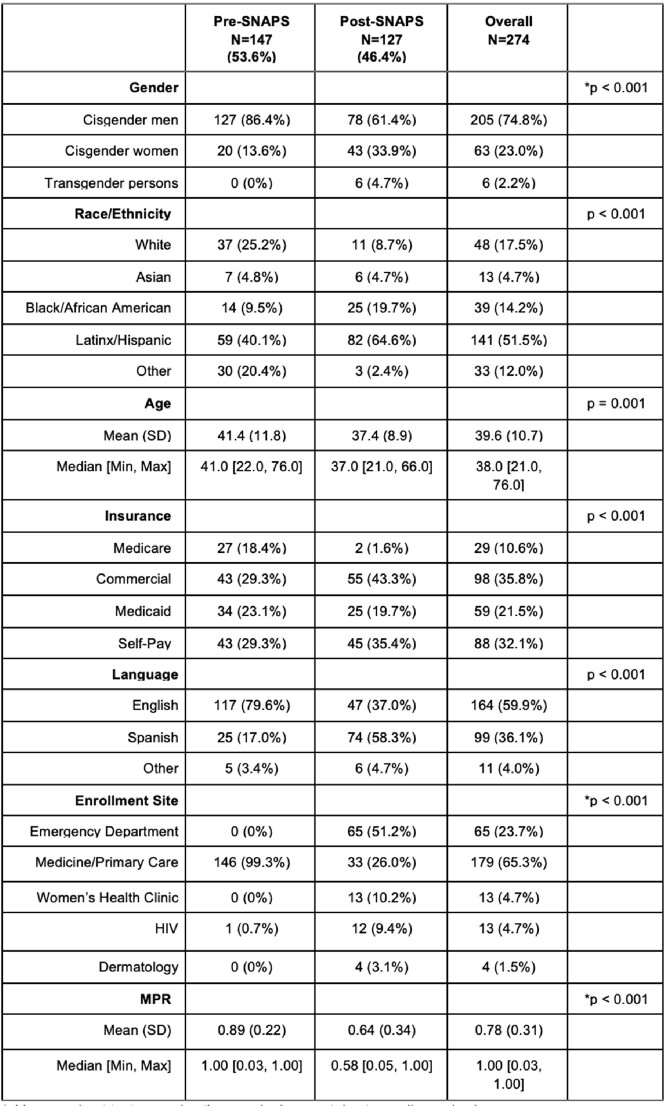
Demographic Profiles For Pre-SNAPS and Post-SNAPS Individuals. Chi-squared or t-test approximation may be incorrect due to small sample size.

**Table 2.**
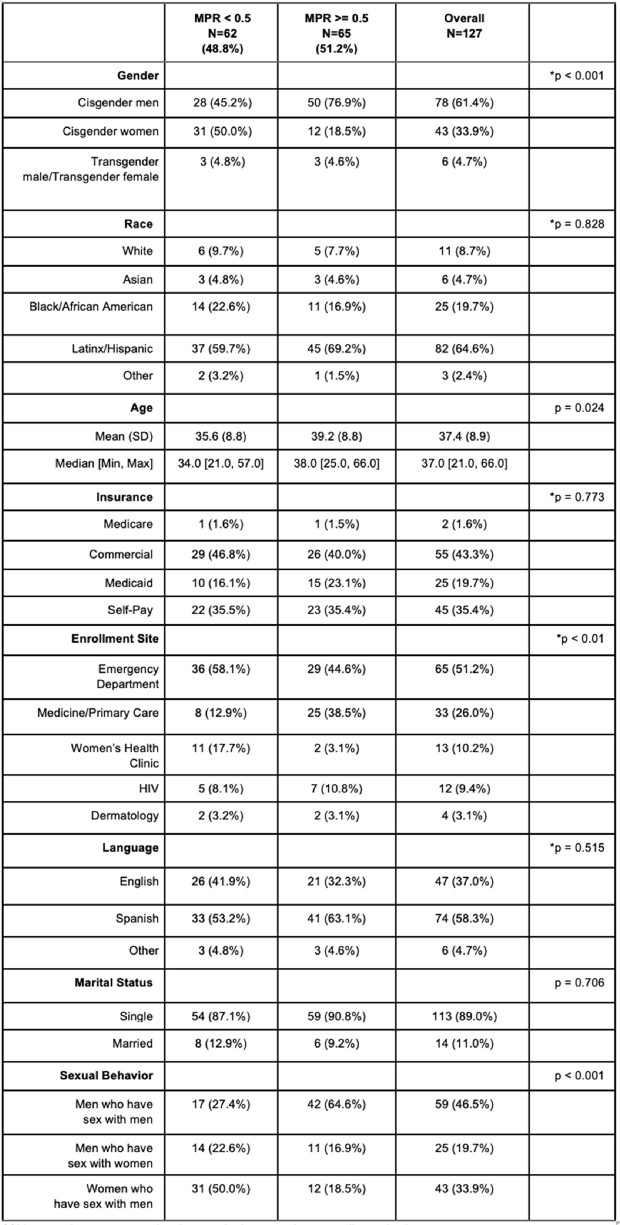
MPR for Post-SNAPS Individuals After 1 Year of Follow-up. Chi-squared or t-test approximation may be incorrect due to small sample size.

**Methods:**

**SNAPS** consisted of 5 components: (1) **Surveillance** of clinical sites where STI testing is high but PrEP use is rare e.g., ED and Women’s Health Clinic (WHC), (2) **N**avigation for PrEP-eligible individuals (3) **A**ccelerated follow-up with PrEP experts, (4) **P**oint-of-care counseling and lab testing, and (5) **S**eamless longitudinal care. SNAPS launched 6/2019 with 2 full-time navigators. One year pre- vs post-SNAPS implementation we compared the sociodemographic profiles of PrEP initiators, their site of enrollment, and their medication possession ratios (MPRs), a proxy for PrEP adherence. Those on PrEP were a mixture of urgent and continuity care adults initiating PrEP at a safety-net hospital system. Bivariable analyses, employing Chi-square and t-test statistics, were conducted to compare sociodemographic profiles and MPR pre- vs post-SNAPS.

**Results:**

We analyzed data on 274 (n=147 pre-SNAPS and n=127 post-SNAPS) individuals on PrEP. Compared to the pre-SNAPS period, post-SNAPS individuals were more likely to be cisgender women (33.9% vs 13.6%), Black or Latinx (84.3% vs 49.6%), uninsured (35.4% vs 29.3%), and Spanish speaking (58.3% vs 17.0%) (Table 1). Post-SNAPS individuals were more likely to be started on PrEP from the ED (51.2% vs 0%) or WHC clinic (10.2% vs 0%). Mean MPR for post-SNAPS was significantly lower than pre-SNAPS, 0.64 vs 0.89 (p < 0.001). Among post-SNAPS individuals, MSM were more likely to have higher MPRs compared to MSW and WSM individuals (Table 2). Furthermore, no difference in MPR was noted by race, preferred language, or insurance type.

**Conclusion:**

SNAPS successfully identified and linked key populations historically missed for PrEP opportunities. Efforts to improve SNAPS should target medication adherence.

**Disclosures:**

**All Authors**: No reported disclosures

